# Novel mixed heterovalent (Mo/Co)O_x_-zerovalent Cu system as bi-functional electrocatalyst for overall water splitting

**DOI:** 10.1038/s41598-024-54934-9

**Published:** 2024-02-26

**Authors:** Ahmed R. Tartour, Moustafa M. S. Sanad, Ibrahim S. El-Hallag, Youssef I. Moharram

**Affiliations:** 1 Central Metallurgical Research and Development Institute, P.O. Box: 87, Helwan, Cairo 11421 Egypt; 2 Electroplating Department, Factory 100, Abu-Zaabal Company for Engineering Industries, Cairo, Egypt; 3 Chemistry Department, Faculty of Science, Tanta University, Tanta, Egypt

**Keywords:** Chemistry, Energy science and technology, Materials science, Materials for energy and catalysis

## Abstract

A novel hybrid ternary metallic electrocatalyst of amorphous Mo/Co oxides and crystallized Cu metal was deposited over Ni foam using a one-pot, simple, and scalable solvothermal technique. The chemical structure of the prepared ternary electrocatalyst was systematically characterized and confirmed via XRD, FTIR, EDS, and XPS analysis techniques. FESEM images of (Mo/Co)O_x_–Cu@NF display the formation of 3D hierarchical structure with a particle size range of 3–5 µm. The developed (Mo/Co)O_x_–Cu@NF ternary electrocatalyst exhibits the maximum activity with 188 mV and 410 mV overpotentials at 50 mA cm^−2^ for hydrogen evolution reaction (HER) and oxygen evolution reaction (OER), respectively. Electrochemical impedance spectroscopy (EIS) results for the (Mo/Co)O_x_–Cu@NF sample demonstrate the minimum charge transfer resistance (R_ct_) and maximum constant phase element (CPE) values. A two-electrode cell based on the ternary electrocatalyst just needs a voltage of about 1.86 V at 50 mA cm^−2^ for overall water splitting (OWS). The electrocatalyst shows satisfactory durability during the OWS for 24 h at 10 mA cm^−2^ with an increase of only 33 mV in the cell potential.

## Introduction

The worldwide strive for renewable energy sources is progressively growing due to the limitations of fossil fuels, environmental pollution, and climate change problems^1^. Therefore, utilizing low-cost, clean, and efficient energy technologies are one of the targets to achieve sustainable developments for future generations^2,3^. Electrochemical energy storage and conversion systems such as fuel cells, batteries, and water electrolyzers are currently promising alternatives for the aforementioned world crisis^4–9^. Actually, all these systems principally depend on renewable resources such as solar energy. Recently, solar energy powered-electrochemical water splitting has been considered the most optimistic technology for large-scale H_2_ fuel production^10,11^. In fact, electrochemical water splitting produces high-purity O_2_ and H_2_ gases with the aid of catalytic electrode surfaces^12^. There are many benchmark catalytic materials that can be used to catalyze hydrogen evolution reaction (HER) such as Pt, Ru, Ir, and other noble metals-based materials^13^. Besides, Ir, Ru, IrO_2_, and RuO_2_ represent the state-of-the-art electrocatalysts used for catalyzing the oxygen evolution reaction (OER) process with high performance^14^. However, such noble metals are expensive resulting in high total costs for energy devices. Therefore, seeking more economical and efficient electrocatalysts is crucial^15–18^. Currently, a vast amount of published articles have been devoted to developing highly efficient electrocatalysts for HER and OER based on non-noble elements. Transition metals and their derived materials such as oxides^19–21^, chalcogenides^22–24^, phosphides^25,26^, nitrides^27^, layered double hydroxides (LDH)^28,29^, and metal–organic frameworks (MOFs)^30,31^ performed as the most applicable materials for catalyzing the water splitting^32^.

Copper and cobalt-based materials were reported as efficient electrocatalysts for water splitting because of their economical price, plenty of supply, unusual coordination chemistry, and varied redox characteristics^33–35^. Cu is one of the most desirable transition metals. Cu-based materials demonstrated a wide range of catalytic performances depending on their structure and composition^34^. For example, the dense Cu_x_O nanowires fabricated on Cu foam achieved good catalytic activity and considerable stability in 1 mol L^−1^ KOH^36^. It showed low overpotential values of about 135 mV and 315 mV at 10 mA cm^−2^ and small Tafel slopes of 135 mV dec^−1^ and 63 mV dec^−1^ for HER and OER, respectively. Additionally, macroporous CoO covered by Co/N-doped graphitic carbon nanosheet arrays revealed enhanced electrocatalytic performance for HER and OER^37^. The symmetric-two electrode cell composed of this electrocatalyst showed a stable cell voltage of 1.62 V at 10 mA cm^−2^ for 35 h. Nevertheless, these monometallic-based materials have some limitations in their electrochemical activities, attributed to the intrinsic charge transfer resistance, scarcity of exposed active centers, and short-term chemical and thermodynamic stability^38^. Lately, plenty of published articles highlighted and investigated the stimulated actions of bi-metallic materials for the water-splitting process^39–41^. Based on this, the binary cobalt/copper-based bimetallic materials indicated an enhanced catalytic behavior towards both HER and OER. Specifically, two-dimensional CuCo_2_O_4_ nanosheets have exhibited high electrocatalytic performance towards both HER and OER, with small overpotential values of 115 and 290 mV at 10 mA cm^−2^, and excellent durability at 1.0 mol L^−1^ KOH^42^. This bi-functional electrocatalyst can deliver a cell voltage of 1.64 V at 10 mA cm^−2^. Moreover, nanoporous CoCu-layered double hydroxide (LDH) was very effective as bifunctional electrocatalyst and showed small overpotential values of 110 and 245 mV at 10 mA cm^−2^ with low Tafel slope values of 46.4 and 32 mV dec^−1^, and excellent durability at 1.0 mol L^−1^ KOH^43^. The electrolysis cell composed of this bifunctional electrocatalyst possessed 1.60 V at 10 mA cm^−2^. More recently, the Mo incorporation into many binary metallic materials greatly boosted the activity and durability of these electrocatalysts toward overall water-splitting^38^.

Accordingly, employing multi-metallic structures or their derivatives could be an effective strategy to obtain high-performance bi-functional catalysts for overall water splitting (OWS)^44,45^. Xin et al. have prepared 3D core–shell CoCu/CuCoMoO_x_ nanosheets decorated with nanoparticles supported on copper foam^46^.They reported that the prepared electrocatalyst achieved low overpotential values of ~ 75 mV and 315 mV at 100 mA cm^−2^ for HER and OER, respectively, and a low voltage of ~ 1.66 V for OWS as well. On the other hand, Mo-doped hybrid Cu/Co oxide prepared by a hydrothermal route displayed strong HER withan overpotential ~ 88 mV at 10 mA cm^−2^ in alkaline electrolyte with high stability for 28 h and long cycle life over 5000 cycles^47^. Another work implemented by Santos et al. revealed that the addition of copper to the electrodeposited bimetallic Co–Mo systems enhanced the catalytic performance towards HER^48^. Precisely, the electrodeposited film of Co_56_Mo_21_Cu_23_ exhibited a lower overpotential at 119 mV compared to that of Co-Mo bimetallic coating Co_67_Mo_33_at 156 mV at 10 mA cm^−2^.

In this work, an electrocatalyst of a novel heterovalent hybrid amorphous and crystalline structure based on the three metals Co, Cu, and Mo altogether was simply fabricated on a nickel foam (NF) surface through a one-step and scalable solvothermal technique. Our work is more advantageous than the aforementioned reported works with respect to method simplicity, energy saving, and electrocatalyst efficiency. Although the electrocatalysts prepared by Xin et al. and Liu et al.^46,47^ are considered as good as our ternary electrocatalyst, their preparation is more sophisticated and passed through many steps including annealing at high temperatures (500 °C, and 300 °C, respectively) under reducing atmosphere. Besides, the electrocatalysts prepared by Santos et al.^48^ were only assessed for HER, showing comparable results to our electrocatalyst. Plainly, the structure and morphology were investigated using scanning electron microscopy (SEM), energy dispersive spectroscopy (EDS), X-ray diffraction (XRD), and X-ray photoelectron spectroscopy (XPS). HER and OER catalytic activity of the ternary electrocatalyst (Mo/Co)Ox–Cu@NF in alkaline medium was highlighted in contrast to the binary (CoO_x_–Cu@NF) and unary electrocatalysts (Cu@NF) under the same conditions. Moreover, the activity and durability towards the OWS using two similar electrodes of (Mo/Co)O_x_–Cu@NF were evaluated. Voltammetry, electrochemical impedance spectroscopy (EIS), and chronopotentiometry techniques were used for the assessment of the activity and durability of the prepared electrocatalysts.

## Experimental

### Electrocatalyst preparation

**Figure 1:**
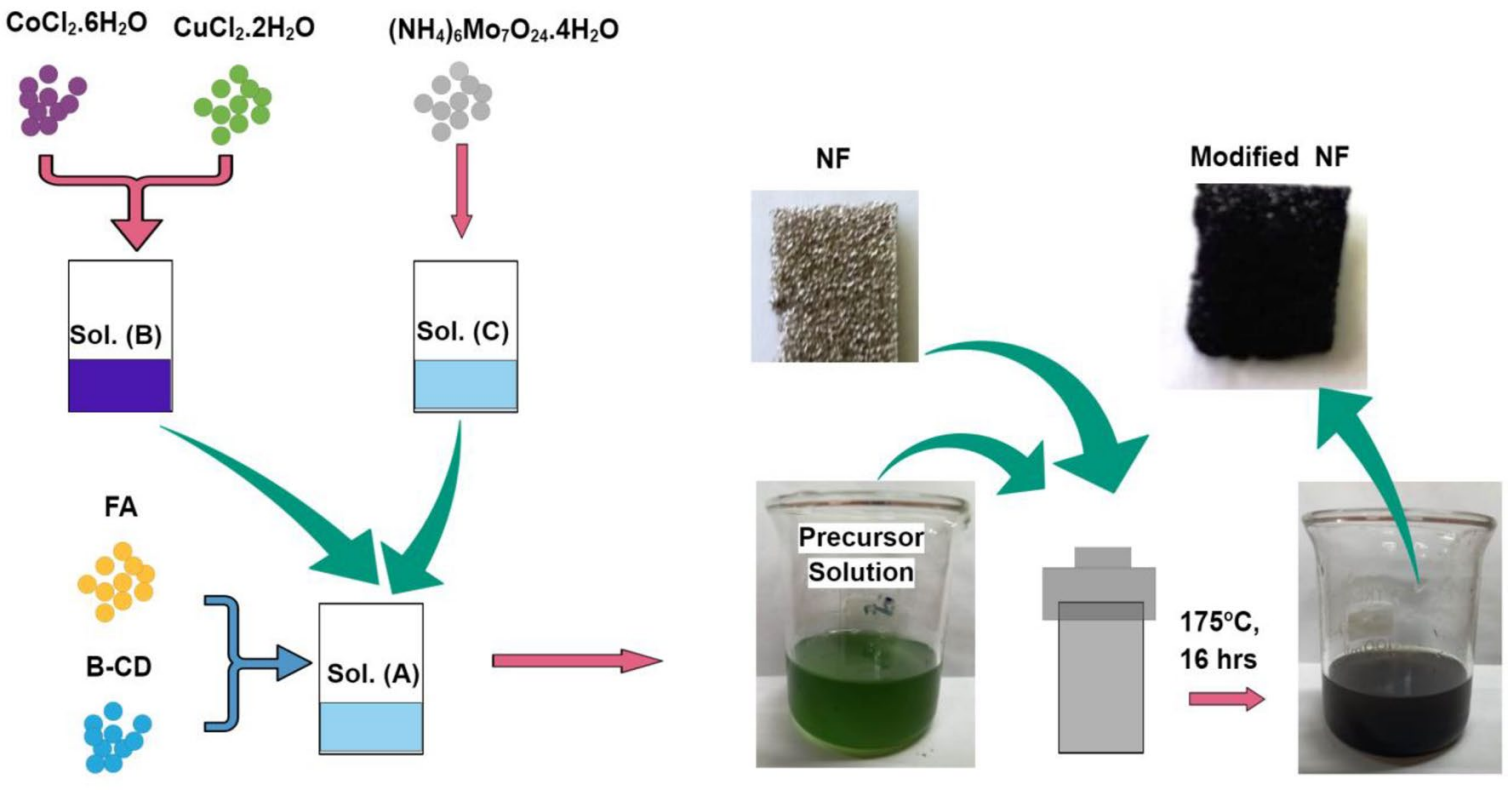
Illustration for the fabrication procedure of (Mo/Co)O_x_–Cu@NF.

The ternary electrocatalyst (Mo/Co)O_x_–Cu@NF was prepared via a solvothermal approach as exhibited in Fig. 1. Firstly, 1 mmol fumaric acid (FA, 99%), and 0.1 mmol β-cyclodextrin (β-CD, 98%) were dissolved in 24 ml Dimethyl formamide (DMF, 99%) to form a clear solution (A). 0.5 mmol CoCl_2_·6H_2_O,and 0.5 mmol CuCl_2_·2H_2_O were separately dissolved in 6 ml absolute ethanol to form a clear solution (B). 0.1 mmol of (NH_4_)_6_Mo_7_O_24_·4H_2_O was also dissolved in 6 ml deionized water to form a clear solution (C).Then, we simultaneously added solutions (B) and (C) into solution (A), and let the mixture under stirring for 1 h. On the other hand, nickel foam (NF) pieces of dimensions (2 cm × 0.5 cm) were cleaned with 6 mol L^−1^ HCl solution for 30 min, followed by cleaning with deionized water and ethanol for 10 min under vigorous shaking. After that, The NF substrates were placed in a 100 ml Teflon-lined stainless steel autoclave, and the prepared Mo–Co–Cu solution mixture was poured into this autoclave. Thereafter, the closed autoclave was heated for 16 h in an electric oven at 175 °C. After cooling the autoclave to room temperature, the ternary coated (Mo/Co)O_x_–Cu@NF substrate was washed with deionized water and ethanol multiple times and then dried at 80 °C for 12 h. For TEM and powder XRD measurements, the same preparation procedure was used but without adding NF in the precursor solution and the product was centrifuged and washed with distilled water and ethanol and dried at 80 °C.

**Figure 2:**
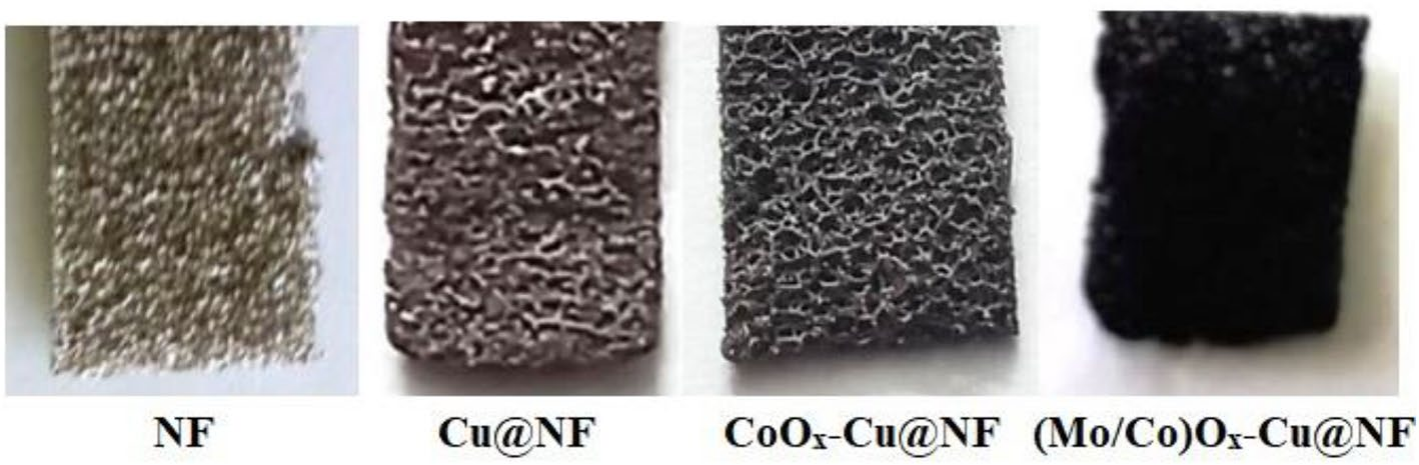
Surfaces of the blank uncoated-NF and the three coated-NF samples.

For comparison, The CoO_x_–Cu@NF and Cu@NF systems besides all the metallic combinations-based catalyst systems were prepared by the same procedure (see supplementary) without the addition of (NH_4_)_6_Mo_7_O_24_·4H_2_O, and (NH_4_)_6_Mo_7_O_24_·4H_2_O/CoCl_2_·6H_2_O salts, respectively. Figure 2 displays the different colors of the prepared samples (Mo/Co)O_x_–Cu@NF, CoO_x_–Cu@NF, and Cu@NF of different compositions. It is clear from the brownish-red color in the Cu@NF sample indicates that zero-valent copper was formed.

### Structure characterization

The texture and morphology of the prepared samples were characterized using a field emission electron microscope (FESEM, QUANTAFEG 250). In addition, energy-dispersive X-ray spectroscopy (EDS) and elemental mapping (EM) integrated with basic FESEM were used for elemental surface analysis. Functional groups and chemical bonds were verified using Thermo Nicolet Fourier-transform infrared spectroscopy (FT-IR). Transmission electron microscope (TEM), high resolution TEM (HR-TEM) images, and the selected area electron diffraction (SAED) pattern were captured to confirm the composite structure using JEOL TEM 2100plus instrument. X-Ray Diffraction (XRD) patterns were recorded using Bruker axis D8 diffractometer with radiation source Cu-Kα (λ = 1.5406 nm) at 40 kV and 30 mA to determine the crystalline phases. X-ray photoelectron spectroscopy (XPS) data were collected with K-alpha Thermo Scientific AXIS 165 (Thermo Fisher Scientific, USA) spectrometer to investigate the oxidation states of the existing elements.

### Electrochemical characterization

A conventional three-electrode electrochemical cell was used for the measurements with 1 mol L^−1^ KOH solution used fresh for each measurement at 25 °C as an electrolyte. Ag/AgCl/3 mol L^−1^ KCl and Pt foil were used as reference and counter electrodes, respectively. The employed active area of the working electrode is 0.4 cm^2^. The active areas of the working and the counter electrodes were kept to be in a constant ratio of (1:2) through all the experiments. BioLogic MPG-205 potentiostat was used to perform the electrochemical measurements. For general electrochemical assessment cyclic voltammetry (CV) measurements were obtained for the fabricated electrodes at the applied potential range (+ 0.90 to − 1.70) V versus Ag/AgCl/3 mol L^−1^ KCl, with a scan rate of 50 mV s^−1^. In addition, linear sweep voltammetry (LSV) data were recorded with scan rate of 5 mV s^−1^ in the ranges (− 0.90 to − 1.70) V and (+ 0.2 to + 0.90) V versus Ag/AgCl/3 mol L^−1^ KCl for HER and OER catalytic studies, respectively. All potential values for the LSVs were converted to be expressed versus the reversible hydrogen electrode (RHE) using the relation:

1 $${{\text{E}}}_{{\text{RHE}}} = {{\text{E}}}_{{\text{Ag}}/{\text{AgCl}}} + \left(0.059\times {\text{pH}}\right) + 0.210$$

where 0.210 is the standard potential of the Ag/AgCl/3 mol L^−1^ KCl veraus RHE at 25 °C. The overpotential values in the volt unit for HER and OER are given by subtracting the standard potential values (0 V for HER and 1.23 V for OER) from experimental potential versus RHE (${{\text{E}}}_{{\text{RHE}}}$). All measurements were corrected for the voltage drop due to the uncompensated resistance (jR_u_-drop) with a compensation percentage of 80% using EIS measurements.

EIS measurements for all fabricated electrodes were implemented at − 0.274 V versus RHE for HER and + 1.806 V versus RHE for OER. EIS data were obtained in the frequency range of 20 kHz to 0.1 Hz, with an alternating AC voltage of 10 mV amplitude. The double-layer capacitance (C_dl_) was obtained from cyclic voltammetry (CV) measurements using different scan rates (from 10 to 100 mV s^−1^) in a non-faradic potential range. The measured capacitive current density (Δj/2 =|j_c_-ja|/2) was plotted versus the scan rates, and the obtained linear slope value was considered equivalent to C_dl_. Where j_c_ and ja are the cathodic and anodic current densities, respectively at the potential values revealed in Fig. 8.

Furthermore, the electrochemical active surface area (EASA) was calculated using the relation.

2 $$\mathrm{EASA }=\frac{{{\text{C}}}_{{\text{dl}}}}{{{\text{C}}}_{{\text{S}}}}$$

where ${{\text{C}}}_{{\text{dl}}}$ and ${{\text{C}}}_{{\text{S}}}$ are the double layer capacitance in mF and specific capacitances in mF cm^−2^, respectively. The widely reported average value of 0.040 mF cm^−2^ was used for the C_S_^49^. Furthermore, the roughness factor (R_f_) is calculated by dividing the EASA on the geometric area (0.4 cm^2^).

For OWS evaluation, the durability measurements for symmetric two-electrode cell were conducted using the chronopotentiometry technique by applying a constant current at 10 mA cm^−2^ for 24 h. Further, In order to evaluate the required voltage for the overall water splitting cell, a LSV was recorded using symmetric two-electrode cell.

## Results and discussion

### Morphology, composition, and structure characterization

Scanning electron microscopic images (SEM) were used to investigate the morphology of the deposited film of unary Cu@NF and ternary (Mo/Co)O_x_–Cu@NF electrocatalysts at different magnifications. Figure S1 in the supplementary data file displays the uniform cubic crystals of deposited zerovalent Cu on Ni foam surface for the unary Cu@NF electrocatalyst.

**Figure 3:**
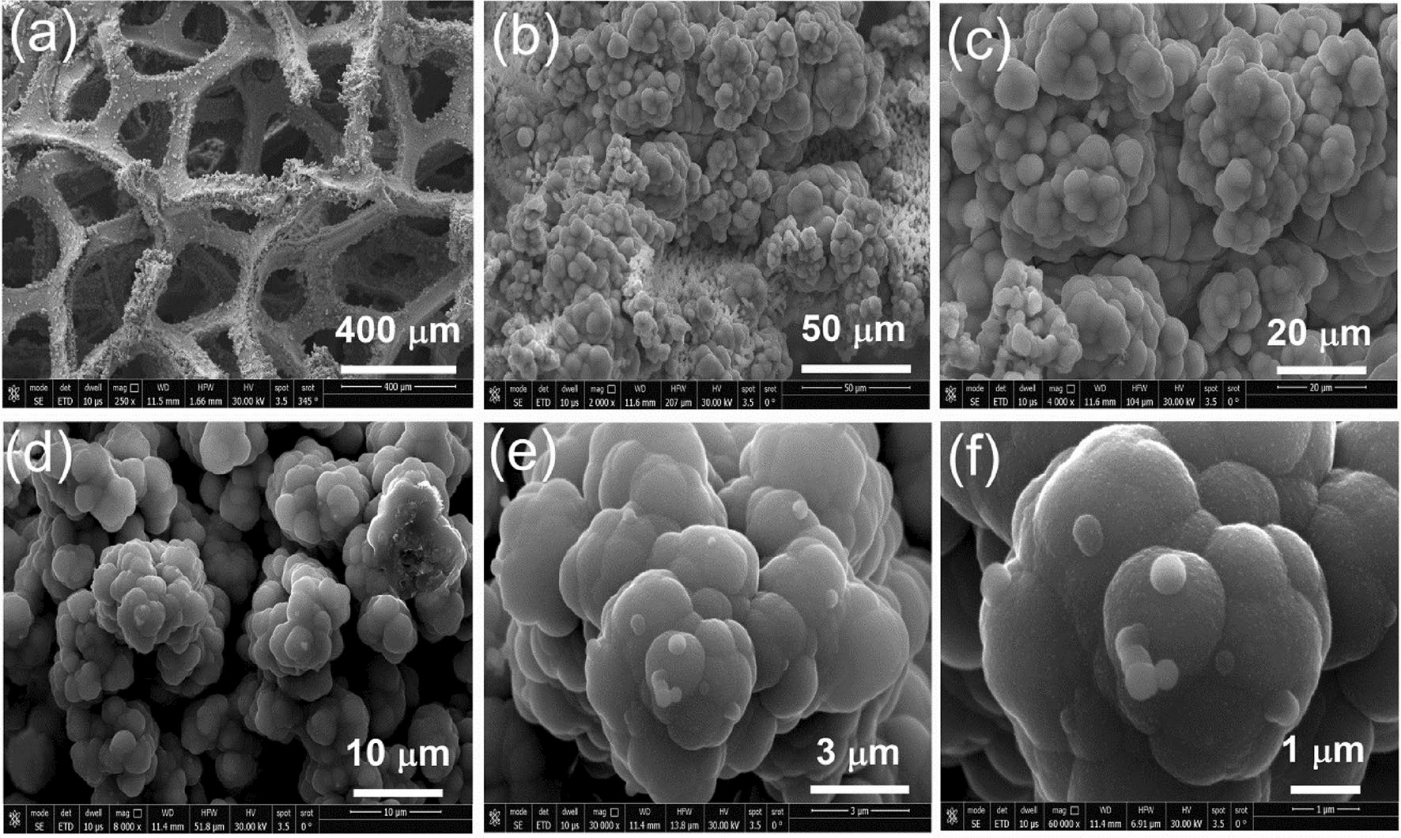
FE-SEM images (**a**–**f**) showing the texture and morphology of the fabricated (Mo/Co)O_x_–Cu@NF at different magnifications.

On the other hand, Figure 3a–f indicate that the (Mo/Co)O_x_–Cu@NF has a rose-type flower hierarchical structure which is characterized by a high surface area. The dimensions of each flower are ranged between 3 and 5 µm.

**Figure 4:**
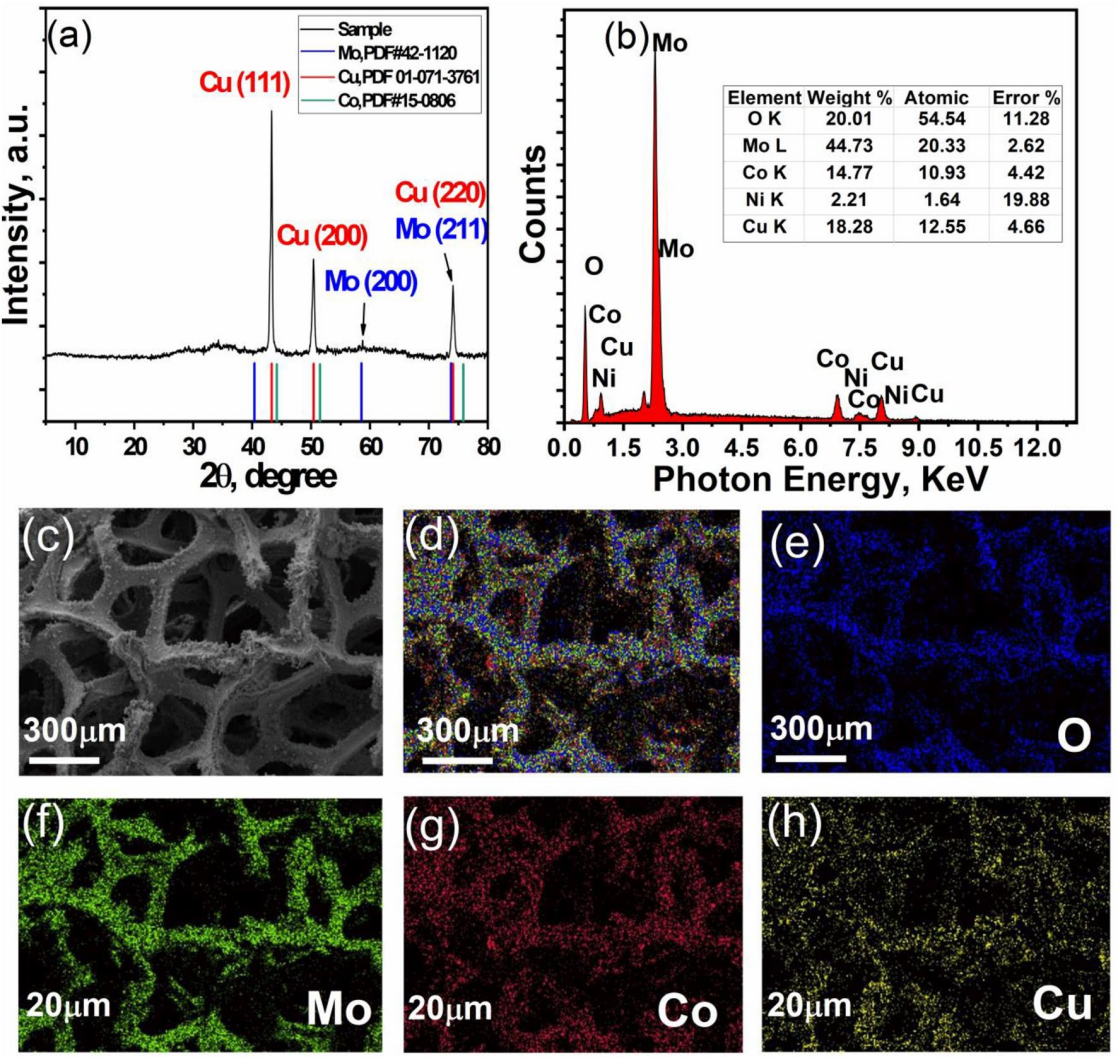
(**a**) XRD pattern of (Mo/Co)O_x_–Cu@NF and (**b**) EDS spectrum for the selected SEM area (**c**) and their corresponding elemental mapping (**d**–**h**).

The X-ray diffraction measurement (Fig. 4a) shows a clear cubic crystalline structure of metallic Cu of space group Fm-3 m which coincides with the standard card PDF#01-071-3761. The average crystallite size was calculated based on the characteristic peaks at 2θ (43.301°, 50.410°, and 74.084°) to be (D_avg_ = 55 nm) using Scherrer equation (Eq. 3):

3 $$D= \frac{K*\lambda }{\beta *{\text{cos}}\theta }$$

where K is the shape factor of value 0.9, β is full width at half maximum of peak (FWHM), λ is the X-ray wavelength of value 1.54 nm, θ is the diffraction angle in radians.

The lattice strain for the main peak (ε = 0.076) is obtained using Eq. 4:

4 $$\varepsilon = \frac{\beta }{4{\text{tan}}\theta }$$

The dislocation density (δ) was estimated based on D_avg_ to be 3.3 × 10^–4^ using Eq. 5:

5 $$\delta = \frac{1}{{D}^{2}}$$

A tiny peak can be detected at 2θ ~ 58.8°, which confirms the presence of Mo phase but the rest of peaks were not observed, which could be related to its non-crystallinity. Moreover, no peaks are observed for cobalt due to the presence in an amorphous phase. EDS technique will confirm the existence of Mo, Co, and Cu elements in the ternary electrocatalyst, as well as the presence of Cu element in the unary electrocatalyst.

EDS analysis of Cu@NF shows the atomic percentage of deposited Cu to be 70.74% and 29.26% for Ni, which is attributed to the Ni foam as shown in Fig. S2b. The corresponding elemental mapping for the total area in SEM image (Fig. S2a) illustrates the homogeneous distribution of the deposited Cu over Ni foam as shown in Fig. S2c–e.

Figure 4b demonstrates the EDS spectrum analysis of the area corresponds to Fig. 3c (also see Fig. S3). The prepared composite film showing atomic percentage of O = 54.54%, Mo = 20.33%, and Co = 10.93%, Cu = 12.55%, Ni = 1.64%. Ni percentage is only attributed to the NF substrate. By exempting the Ni percentage, the new atomic percentages will be O = 55.44%, Mo = 20.67%, and Co = 11.11%, and Cu = 12.76%. So the ratio of O: Mo is almost (2.5:1), Co: Cu is around (1:1) and Mo: Co or Mo: Cu are approximately (1.7:1). The homogeneous elemental distribution demonstrated in Fig. 4d–h obtained by the elemental mapping for the selected area shown in Fig. 4c, confirms the formation of a stable and homogeneous chemical structure and not a simple physical mixture.

Further, TEM images confirmed that the ternary catalyst has heterostructure of crystalline metallic copper nanoparticles in a scale of 50 nm imbedded within the amorphous Mo/Co mixed oxide as shown in Fig. S4a. The crystalline structure of copper is indicated by the lattice fringes presented in HR-TEM image (Fig. S4b) which is further confirmed by the bright spot and diffraction rings of SAED image (Fig. S4c) which are characteristic for polycrystalline materials.

**Figure 5:**
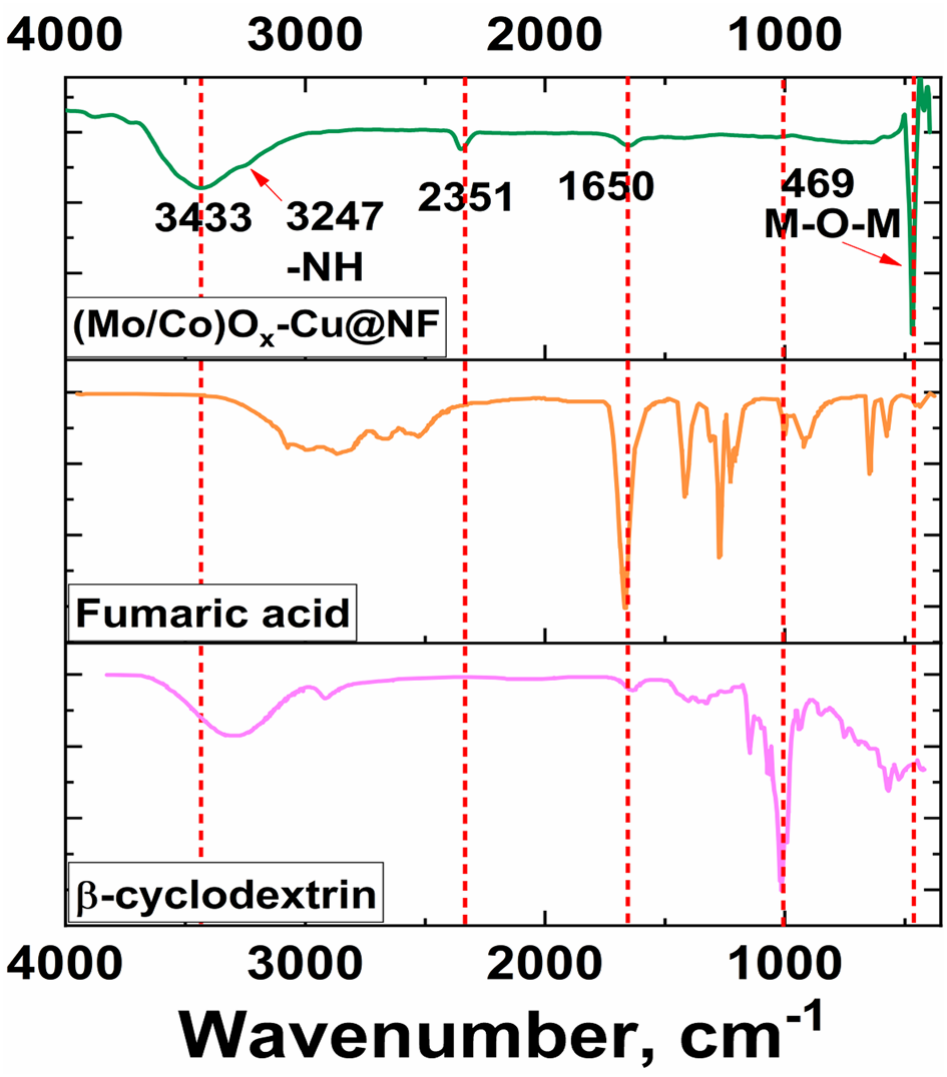
ATR-FTIR for the fabricated (Mo/Co)O_x_–Cu@NFand compared to that of the Fumaric acid, and β-cyclodextrin.

Figure 5 reveals the ATR-FTIR spectra of the prepared ternary system (Mo/Co)O_x_–Cu and the employed organic components in the reaction mixture (fumaric acid, and β-cyclodextrin). The appearance of a sharp peak at 469 cm^−1^ within the characteristic region of the M–O–M bond confirming the presence of Co and Mo oxides in the prepared ternary system^50,51^. The small peak at 1650 cm^−1^ may be attributed to O–H bending of adsorbed water^52^ and C=O stretching of carboxylate group of fumaric acid or the organic residuals produced from oxidative degradation of β-cyclodextrin confirming their action as complexing and reducing agents^53^. Moreover, the disappearance of ether C–O peak at 1020 cm^−1^ β-cyclodextrin is an evidence for its absence in the structure of the fabricated ternary electrocatalyst. Besides, the broad band at 3433 cm^−1^ can be attributed to O–H stretching of adsorbed water^52^ and alcoholic O–H of organic residuals. Also, the small shoulder at 3247 cm^−1^ is probably to the stretching of –N–H bond which is coming from DMF degradation at the high temperature. Small band observed at 2351 cm^−1^ can be ascribed to C–O bond in CO_2_ adsorbed on the surface of the sample from the atmosphere^52^.

**Figure 6:**
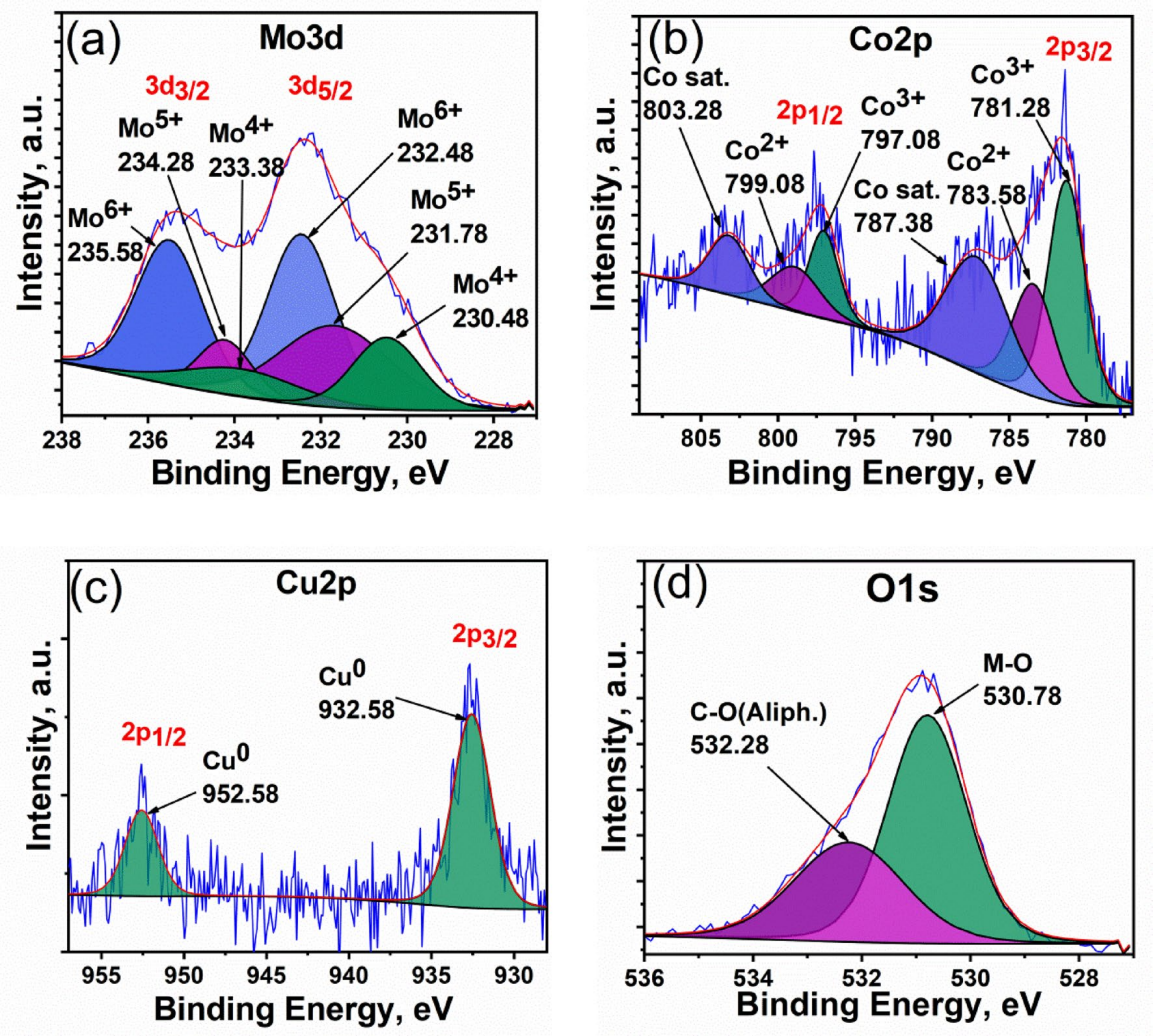
Detailed XPS spectra of (**a**) Mo3d, (**b**) Co2p, (**c**) Cu2p, and (**d**) O1s.

The overall XPS survey presented in Fig. S5a shows the corresponding peaks to different elements existed in the prepared ternary composite and their atomic percentages. The evolution of Cu2p, Co2p, Mo2p, and O1s peaks assures the results obtained by EDS. The appearance of C1s and N1s may be attributed to the organic matrix and DMF solvent used in the preparation. C1s spectra in Fig. S5b describe the bonding types with carbon atoms in the organic residuals. Obviously, three consecutive peaks are detected around 284.88, 286.28, and 288.48 eV as a result of (C–C, C–H), (C–OH), and (O–C=O) bonds, respectively^54^. Ni2p spectra (Fig. S5c) is related to the formation of Ni(OH)_2_ by the partial dissolution of NF in the alkaline medium. Based on the inset table in Fig. S5a including the atomic percentages and by exempting the Ni, N, C percentages, the new atomic percentages will be O = 63.77%, Mo = 28.58%, and Co = 4.19%, and Cu = 3.45%. Generally, we noticed, these percentages are close to that obtained by EDS except for lower percentages of Co and copper. However the ratio between them is approximately the same for O: Mo which is almost (2.5:1) and Co: Cu is around (1:1), the ratios of Mo: Co or Mo: Cu are larger and of approximately (7:1). This slight difference can be explained based on the difference in penetration power of the two techniques through surface of the measured sample, where XPS have lower penetration than EDS, so this means that the surface concentration of Co and Cu oxides is lower compared to the bulk of the catalyst structure. Figure 6a observes the XPS spectra of Mo3d which has characteristic Mo3d_5/2_ peaks with binding energies at (230.48 eV) for Mo^4+^, (231.78 eV) for Mo^5+^, and (232.48 eV) for Mo^6+^ and their corresponding Mo3d_3/2_ peaks with binding energies at (233.48 eV) for Mo^4+^, (234.28 eV) for Mo^5+^, and (235.58 eV) for Mo^6+^^55,56^. The analyzed Co2p spectra (Fig. 6b) show two Co2p_3/2_ peaks around 781.28 and 783.58 eV, and the related satellite at 787.38 eV, characteristic of Co^3+^and Co^2+^, respectively^57^. Alongside, two Co2p_1/2_ peaks with binding energies 797.08 eV and 799.08 eV, correspond to Co^3+^and Co^2+^, respectively, and the related satellite at 803.28 eV^58^. Figure 6c shows the Cu2p spectra with the characteristic Cu2p_3/2_ and Cu2p_1/2_ peaks around 932.58 and 952.58 eV with no satellite peaks which confirms the zerovalent state of Cu^59^. Figure 6d depicts the O1s spectra which can be fitted into two peaks around 530.78 and 532.28 eV. The most intense peak with binding energy ~ 530.78 eV is simply related to oxygen combined with a metal, which indicates the presence of mixed oxide from Mo and Co^55,59,60^, while the other O1s peak at lower binding energy is assigned to aliphatic C–O bonding from organic residuals^61^. The XPS analysis outcomes show high consistency with XRD and EDS results, confirming the formation of the ternary catalytic system.

### Suggested mechanism for the solvothermal synthesis

β-cyclodextrin works as both a reducing and stabilizing agent^53,62–64^, while fumaric works as a complexing and capping agent^65^. At initial stages of reaction, Cu^2+^, Co^2+^, and Co^3+^ ions form complexes with β-CD and FA^66^. The initially introduced Co^2+^ in the reaction mix is easily oxidized to Co^3+^ specially in presence of strong ligating agents under air atmosphere^67^. Then, at high temperature and pressure inside the autoclave, copper is susceptible to being reduced easily by β-cyclodextrin (β-CD) where the primary –CH_2_–OH of β-cyclodextrin is oxidized to be –COOH^53^. Fumaric acid (FA) plays an important role in capping the formed metallic copper and preventing its oxidation. It is proofed from their corresponding standard reduction potentials (E°) which are + 0.34 V for Cu^2+^ versus − 0.28 V for Co^2+^^68^.Co^2+^ ions are not easy to be reduced into the zerovalent state like Cu^2+^ ions in such moderate conditions using a weak reductant (β-CD). Thereby, it is found in its two forms of oxides CoO, and Co_2_O_3_. At the same time, (NH_4_)_6_Mo_7_O_24_·4H_2_O slowly decomposes into molybdenum oxide (MoO_3_), ammonia, and water^69^. A portion of MoO_3_ is reduced into Mo_2_O_5_ and MoO_2_. At the end of the reaction, most of the organic matter is degraded except for some carbon residuals that are attached to the structure. The following equations simply describe the suggested mechanism for the whole solvothermal synthesis.

6 $${\upbeta } - {\text{CD}} + {\text{FA}} + {\text{CuCl}}_{2} \cdot 2{\text{H}}_{2} {\text{O}} \to {\text{Cu}}^{2 + } - {\text{complex}}\mathop{\longrightarrow}\limits_{{{\text{Pressure}}}}^{\Delta }{\text{Cu}}^{0}$$

7 $$\left( {{\text{NH}}_{4} } \right)_{6} {\text{Mo}}_{7} {\text{O}}_{24} \cdot 4{\text{H}}_{2} {\text{O}}\mathop{\longrightarrow}\limits_{{{\text{Pressure}}}}^{\Delta }7{\text{MoO}}_{3} + 2{\text{NH}}_{3} + 7{\text{H}}_{2} {\text{O}}$$

8 $${\upbeta } - {\text{CD}} + {\text{FA}} + {\text{MoO}}_{3} \mathop{\longrightarrow}\limits_{{{\text{Pressure}}}}^{\Delta }{\text{Mo}}_{2} {\text{O}}_{5} {\text{ + MoO}}_{2}$$

9 $${\upbeta } - {\text{CD}} + {\text{FA}} + {\text{CoCl}}_{2} \cdot 6{\text{H}}_{2} {\text{O}} \to \mathop + \limits_{{{\text{Co}}^{3 + } - {\text{complex}}}}^{{{\text{Co}}^{2 + } - {\text{complex}}}} \mathop{\longrightarrow}\limits_{{{\text{Pressure}}}}^{\Delta }{\text{CoO + Co}}_{2} {\text{O}}_{3}$$

### Electrochemical behavior

**Figure 7:**
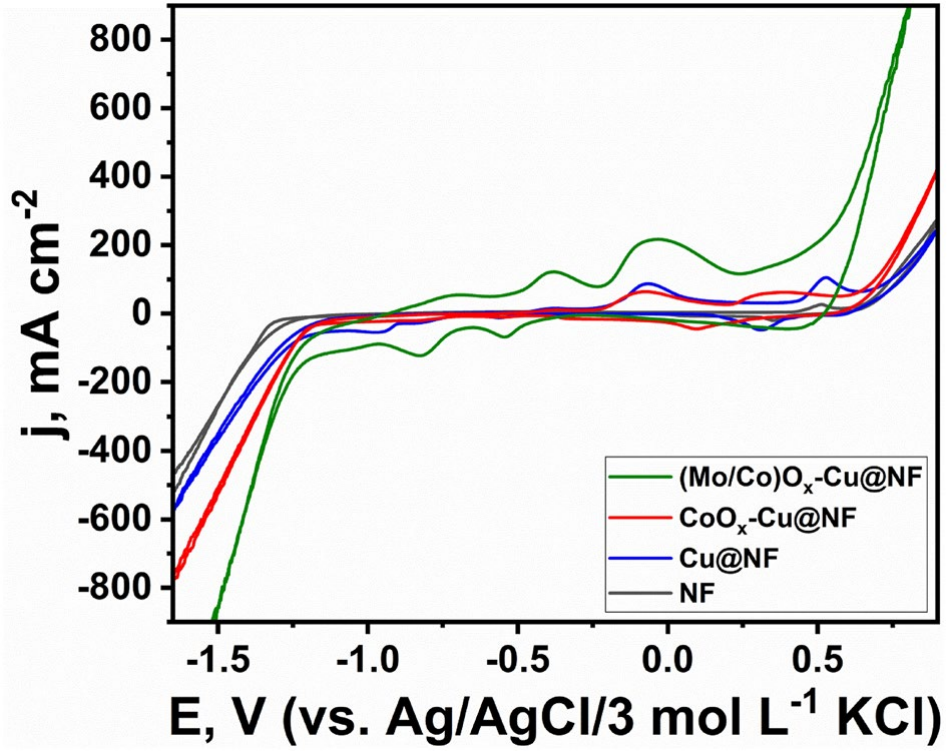
Cyclic voltammetry curves of different samples in 1 mol L^−1^ KOH at scan rate of 50 mV s^−1^ at 25 °C.

Figure 7 compares the cyclic voltammograms (CVs) of Cu@NF, CoO_x_–Cu@NF, and (Mo/Co)O_x_–Cu@NF in 1 mol L^−1^ KOH at a scan rate of 50 mV s^−1^ at 25 °C. NF electrode showed the characteristic peaks of the oxidation (Ni^2+^ to Ni^3+^) at 0.5 V (vs. Ag/AgCl/3 mol L^−1^ KCl) and its corresponding reduction peak at 0.36 V ^70^. Cu@NF electrode exhibits the three anodic peaks at − 0.39 V, − 0.06 V, and 0.53 V corresponding to (Cu^0^ to Cu^1+^), (Cu^1+^ to Cu^2+^), and (Cu^2+^ to Cu^3+^) and their corresponding cathodic peaks at 0.31 V (Cu^3+^ to Cu^2+^), − 0.56 V (Cu^2+^ to Cu^1+^) and − 0.8 V (Cu^1+^ to Cu^0^)^71^. CoO_x_–Cu@NF electrode gives anodic peaks at − 0.39 V corresponding to (Cu^0^ to Cu^1+^), broad peak at − 0.08 V corresponding to both (Cu^1+^ to Cu^2+^) and (Co^2+^ to Co^3+^), and broad peak at 0.34 V corresponding to (Co^3+^ to Co^4+^). The corresponding cathodic peaks are at 0.10 V (Co^4+^ to Co^3+^), − 0.34 (Co^3+^ to Co^2+^), shallow peak at − 0.55 V (Cu^2+^ to Cu^1+^) and − 0.8 V (Cu^1+^ to Cu^0^)^71,72^. Moreover, (Mo/Co)O_x_–Cu@NF electrode gives anodic peaks at − 0.7 V corresponding to (Mo^4+^ to Mo^5+^), − 0.38 V attributed to (Mo^5+^ to Mo^6+^)^73^, and (Cu^0^ to Cu^1+^), and − 0.04 V attributed to (Cu^1+^ to Cu^2+^), and (Co^2+^ to Co^3+^). The peaks at − 0.54 V (Cu^2+^ to Cu^1+^), − 0.82 V (Mo^6+^ to Mo^5+^), and (Cu^1+^ to Cu^0^), and − 1.0 V (Mo^5+^ to Mo^4+^)^73^, represent the corresponding cathodic peaks, respectively. So, it is clear that there are plenty of active centers available for electrocatalyzing either HER or OER.

**Figure 8:**
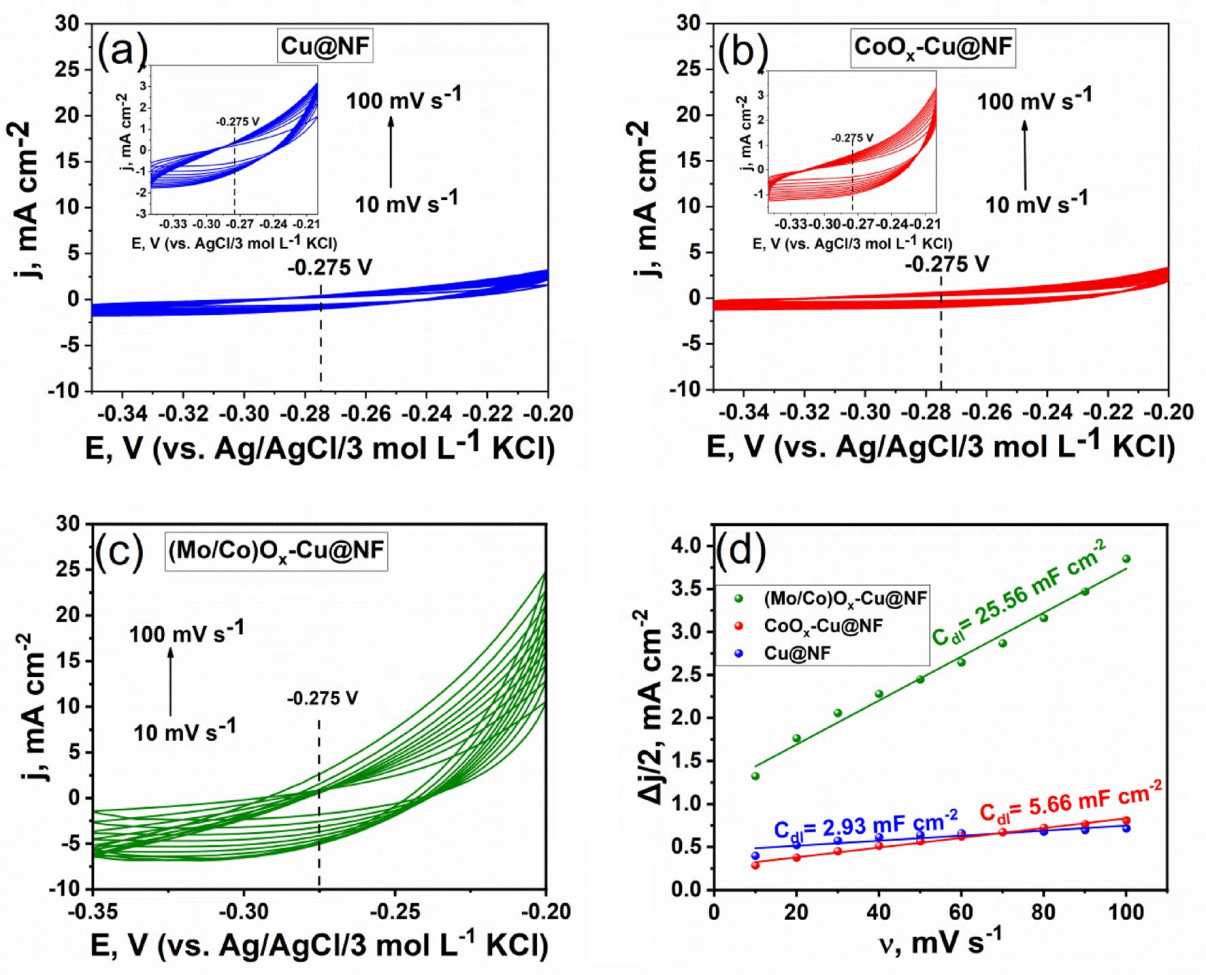
(**a**), (**b**), and (**c**) Cyclic voltammetric curves of different samples swept at different scan rates (10–100 mV s^−1^) in 1 mol L^−1^ KOH, at 25 °C and their corresponding (**d**) plot Δj/2 versus sweep rate.

Furthermore, it is apparent that with increasing the number of elements in the mixed system, the area under the CV curve increases implying an increase in the surface area, and the potential window decreases implying lower over potential for overall water splitting. This behavior can be simply quantified by determining the electrochemical active surface areas (EASA) and the corresponding roughness factor (R_f_). Accordingly, EASA and R_f_ of all samples were calculated according to Eq. 2. from their corresponding double layer capacitance (C_dl_) estimated by cyclic voltammetric curves swept in the non-faradaic processes region at different rates as shown in Fig. 8. Noticeably, the ternary (Mo/Co)O_x_–Cu@NF exhibited the highest capacitance current and the largest active surface area among all electrocatalyst samples, as depicted in Table 1. Moreover, the R_f_ value for the developed ternary electrocatalyst exceeds those values of binary and unary electrocatalysts by more than 4 and 8 times, respectively. Such great enhancement in the EASA of the ternary electrocatalyst could be explained by the presence of mixed Mo/Co-oxides in the amorphous phase which massively improve the surface area and increase the number of active sites. Thus, a higher rate of OER and HER could be obtained at minor increase in overpotential.

**Table 1:** Data extracted from the relation between charging current versus scan rate for each of the prepared electrocatalysts.

Active material	C_dl_ mF cm^−2^	C_dl_ mF		EASA cm^2^	R_f_
(Mo/Co)O_x_–Cu@NF	25.6	10.2		256	639
CoO_x_–Cu@NF	5.66	2.26		56.5	141
Cu@NF	2.93	1.17		29.3	73.3

### Electrocatalytic performance towards HER

**Figure 9:**
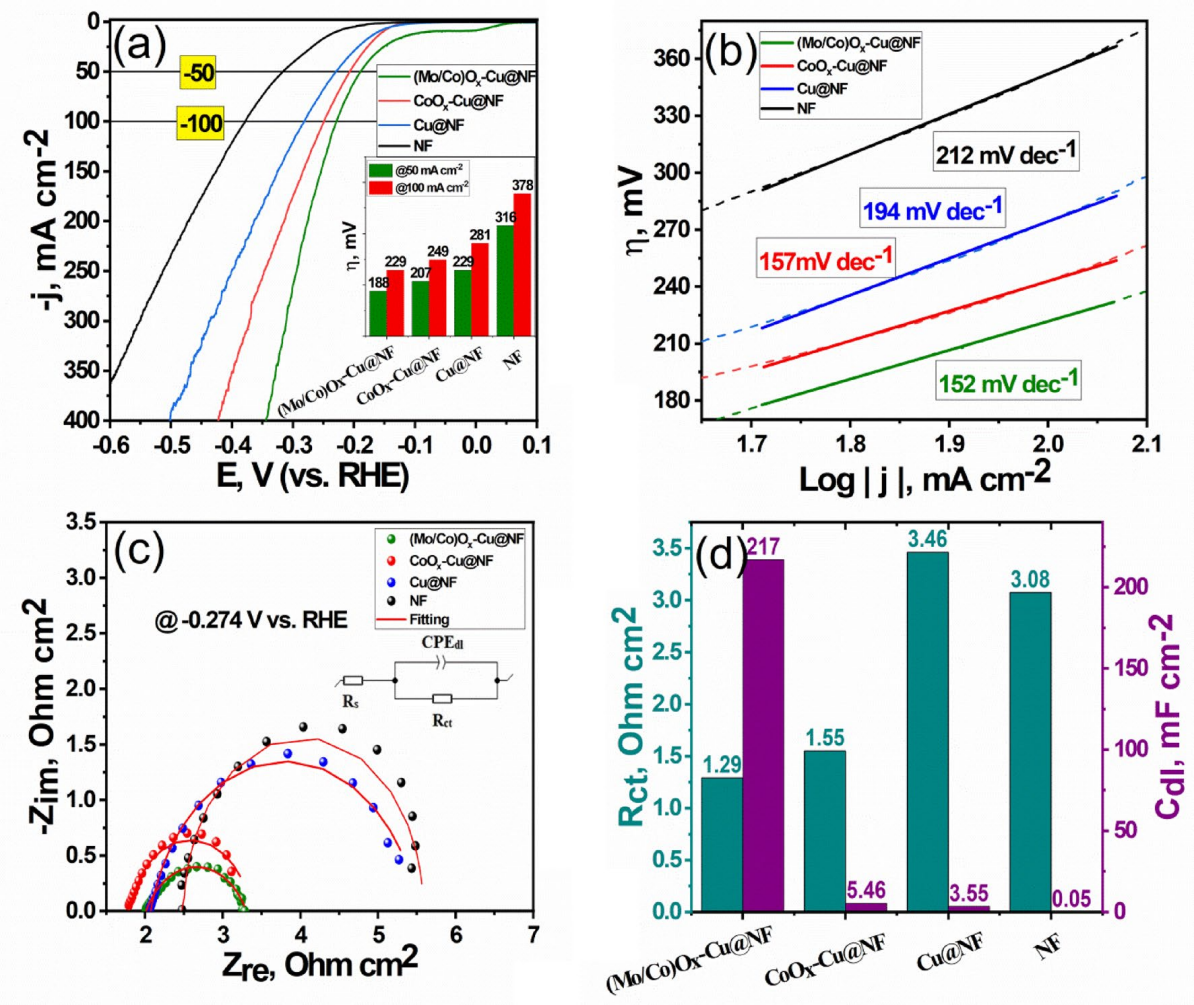
(**a**) Linear sweep voltammetric curves and their corresponding (**b**) Tafel plots for the various prepared samples (**c**) Nyquist plots for different prepared samples measured at − 0.274 V (vs. RHE). (**d**) Graph showing R_ct_ and C_dl_ extracted from EIS for all samples. All measurements recorded in 1 mol L^−1^ KOH, at 25 °C.

The electrocatalytic activities of the ternary (Mo/Co)O_x_–Cu@NF compared to the binary CoO_x_–Cu@NF and Cu@NF towards the HER were measured using a three-electrode system in a standard alkaline medium of 1 mol L^−1^ KOH. Figure 9a shows LSV curves of the NF, Cu@NF, CoO_x_–Cu@NF, and (Mo/Co)O_x_–Cu@NF samples. At the current density of − 50 mA cm^−2^, the overpotential (η) of the (Mo/Co)O_x_–Cu@NF is 188 mV, which is lower than that of the CoO_x_–Cu@NF (207 mV), Cu@NF (229 mV), and NF (316 mV). Figure 9b indicates the corresponding Tafel slope for each catalyst sample. It can be noted that the lowest Tafel slope value was achieved for (Mo/Co)O_x_–Cu@NF (152 mV dec^−1^) compared with the other catalytic combinations. This value of the Tafel slope is close to 120 mV dec^−1^, indicating that HER occurs via the Volmer–Heyrovsky mechanism (Eqs.10, 11)^74^.

Volmer–Heyrovsky mechanism:

10 $${S+H}_{2}O + {e}^{-} \to S-{H}_{ads} + {OH}^{-} (\mathrm{Volmer step})$$

11 $${S-H}_{ads} + {H}_{2}O + {e}^{-} \to {S+ H}_{2} + {OH}^{-} ({\text{Heyrovskystep}})$$

On the other hand, electrochemical impedance spectroscopy (EIS) was recorded for each sample at − 0.274 V versus RHE as shown in Fig. 9c. The impedance spectra recorded were modeled using the modified Randles equivalent circuit comprising from one circuit of CPE_dl_||R_ct_ connected in series with bulk solution resistance (R_s_).

It is observed that Nyquist plot of (Mo/Co)O_x_–Cu@NF has the lowest diameter and lowest height indicating the lowest charge transfer resistance (R_ct_) and the highest constant phase element (CPE_dl_) values compared with the other samples (see Table S1) indicating its high surface area. Figure 9d, and Table 2 display the fitting values of charge transfer resistance (R_ct_), and the effective double layer capacitances (C_dl_) calculated using fitting data presented in Table S1 with the aid of Eq. 12^75,76^ of the different samples.

12 $${{\text{C}}}_{{\text{dl}}}={{{\text{Y}}}_{{\text{dl}}}}^{1/{\text{n}}}\times {{{{({\text{R}}}_{{\text{s}}}}^{-1}+{\text{R}}}_{{\text{ct}}}^{-1})}^{({\text{n}}-1)/{\text{n}}}$$

where Y_dl_ is the CPE_dl_ parameter and n is a dimensionless CPE_dl_ exponent ranging from 0 to 1. Pure capacity is represented by the CPE when n = 1. This equation is more suitable to be used when R_ct_ is lower or comparable to R_s_. It is clearly observed that (Mo/Co)O_x_–Cu@NF has the lowest values for R_ct_ (1.29 Ω cm^2^) and the highest values for C_dl_ (217 mFcm^−2^) compared with those values of the other samples. This confirms the faster charge transfer kinetics and high surface area offered by the heterogeneity of the ternary catalytic system either between amorphous and crystalline phases or between the multiple valences of Co/Mo cations and zerovalent Cu metal. Therefore, one can say that the fabricated (Mo/Co)O_x_–Cu@NF composite demonstrates the best electrocatalytic behavior for HER. Despite the known low intrinsic activity of metallic Cu for catalyzing the water splitting, the obtained results from LSV curves for electrocatalytic systems indicate that the metallic Cu played an important role beside the Co oxide where CoO_x_–Cu@NF and Cu@NF give the highest HER activity after the ternary catalyst (see Fig. S6a,c). Also, the effect of the metallic copper is clear since its addition to the (Mo/Co)O_x_ lowers the (η) value from 309 to 188 mV at 50 mA cm^−2^. This effect may be explained by the surface electron richness and high conductivity of metallic Cu^77^ versus the other individual Mo and Co oxides. In spite of relative high (η) value of (Mo/Co)O_x_@NF electrode, it exhibits high kinetics similar to the ternary catalyst as indicated by apparent investigation of the slopes of LSV curves. This explained by the high intrinsic catalytic activity of these oxides. Concisely, using multimetallic system is effective and promising due to the produced synergistic effect.

**Table 2:** Electrocatalytic parameters towards HER extracted from LSV, Tafel plot, and EIS for the prepared catalytic materials.

Active material	η_50_ (mV)	Tafel slope (mV dec^−1^)	R_ct_ Ω cm^2^	C_dl_ mF cm^−2^
(Mo/Co)O_x_–Cu@NF	188	152	1.29	217
CoO_x_–Cu@NF	207	157	1.55	5.46
Cu@NF	229	194	3.46	3.55
NF	316	212	3.08	0.05

### Electrocatalytic performance towards OER

**Figure 10:**
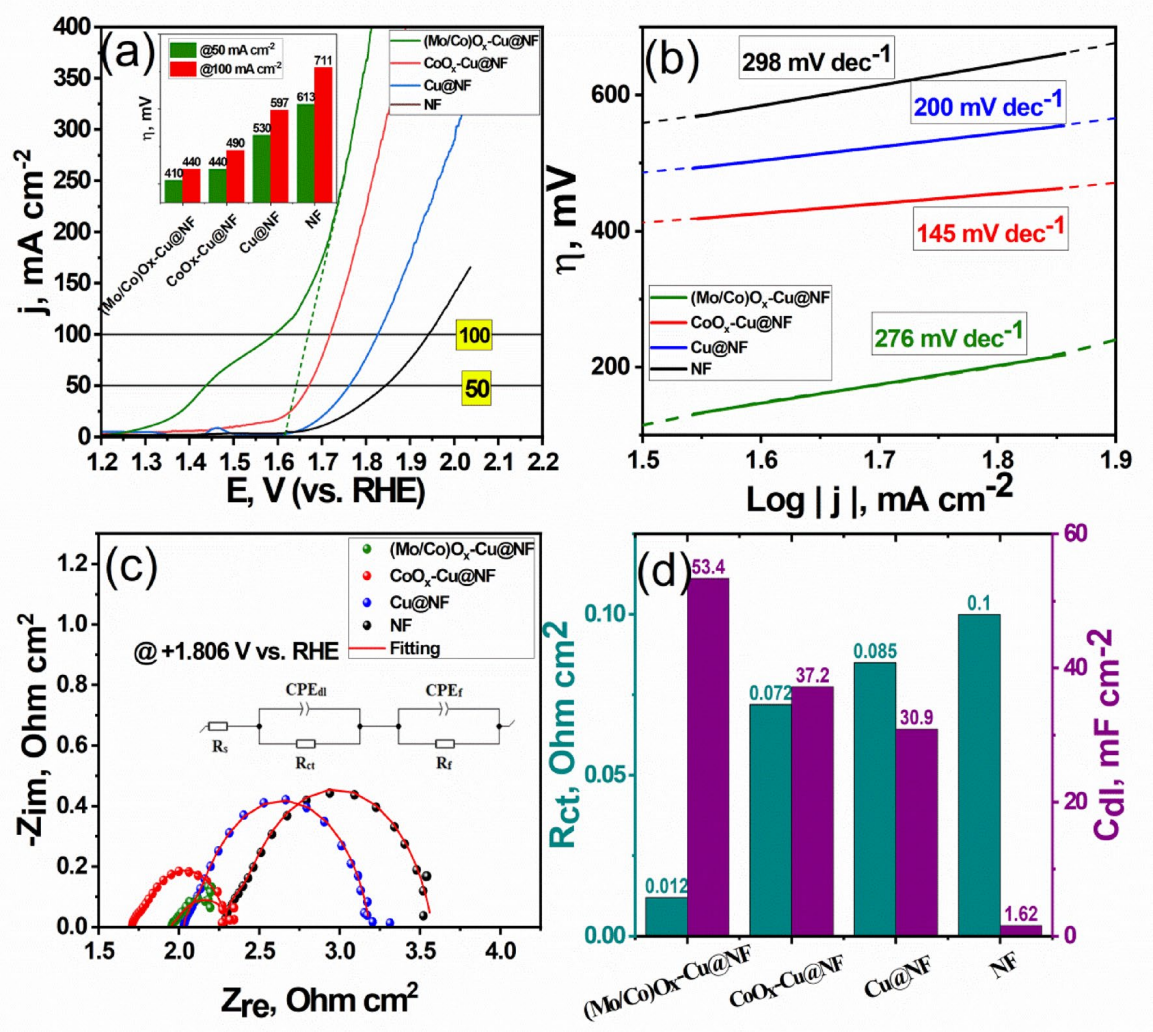
(**a**) Linear sweep voltammetric curves and their corresponding (**b**) Tafel plots for the various prepared samples (**c**) EIS Nyquist plots for different prepared samples measured at + 1.806 V (vs. RHE). (**d**) Graph showing R_ct_ and C_dl_extracted from EIS for all samples. All measurements recorded in 1 mol L^−1^ KOH, at 25 °C.

The electrocatalytic activities of the prepared electrocatalysts for OER were also tested using a three-electrode system in a standard alkaline medium of 1 mol L^−1^ KOH. Figure 10a shows LSV curves of the NF, Cu@NF, CoO_x_–Cu@NF, and (Mo/Co)O_x_–Cu@NF samples. At the current density of 50 mA cm^−2^, the overpotential (η) of the (Mo/Co)O_x_–Cu@NF was 410 mV, which is lower than that of the CoO_x_–Cu@NF (440 mV), Cu@NF (530 mV), and NF (613 mV). Despite the sluggish kinetics of OER over ternary (Mo/Co)O_x_–Cu@NF surface as indicated by the Tafel slope (276 mV dec^−1^) which is of higher value than that of the other two systems (Fig. 10b), it still gives better performance with respect to energy demand since it has the lowest η value toward OER for the same current density^74^. The peak in Fig. 10a preceding the sharp increase in current that corresponds to OER could be attributed to Cu^2+^ to Cu^3+^, Co^3+^ to Co^4+^, or Mo^5+^ to Mo^6+^. The produced high-valence oxides (HVOs) may contribute to increasing the catalytic activity towards OER. This is because metal sites with a higher oxidation state possess optimized e_g_ orbital filling which keeps the binding energy between the oxygen intermediates and the catalytic sites in balance. Furthermore, the OER can be accelerated by surface metal cations and adsorbates transferring charges thanks to the great covalency of M–O in HVOs. Moreover, HVOs promotes the more efficient lattice oxygen-mediated mechanism (LOM) pathway overcoming the drawback of the adsorbate evolution mechanism (AEM)^78^. So The thermodynamic enhancement of the catalytic effect of (Mo/Co)O_x_–Cu@NF toward OER could be attributed to several reasons; (i) the presence of multiple valences of Cu, Co, and Mo cations which enhance the exposed active centers and (ii) the high surface area of the amorphous Mo oxides attained by adding Mo to the bimetallic system CoO_x_–Cu/NF^15,74,79^ which is implied from the high roughness factor (R_f_) (iii) The enhancement of charge transfer originated from the metallic copper and the heterovalent Mo and Co-oxides (iv) The high intrinsic activity of Co oxides towards OER as implied by Tafel slope due to its well-reported optimum adsorption free energy of the OER intermediates.

Further, EIS spectra were recorded for each sample at + 1.806 V versus RHE as shown in Fig. 10c. The Nyquist plot indicated that the ternary electrocatalyst (Mo/Co)O_x_–Cu@NF has the smallest semicircle diameter compared with those values of other samples. The impedance spectra of samples were fitted by the so-called 2 CPE model comprising of two CPE||R circuits connected in series to each other and to R_s_ as shown in Fig. 10c. The first circuit CPE_dl_||R_ct_ represents OER kinetics involving the OH^-^ adsorption and charge transfer. The circuit CPE_f_||R_f_ can represent the impedance generated from the further formation of oxide film on the catalyst surface^80,81^. This can be explained by oxidation of metallic copper through the formation of the intermediates, such as MOH, which can be oxidized to MO during OER. In addition, the transition of Co, Mo oxides of lower oxidation states to higher oxidation states. Figure 10d, and Table 3 introduce the estimated values of charge transfer resistance (R_ct_), and the effective double layer capacitance (C_dl_) for each sample calculated using the EIS fitting data introduced in Table S2 according to Eq. 13^81^.

**Table 3:** Electrocatalytic parameters towards OER extracted from LSV,Tafel plot, and EIS for the prepared catalytic materials.

Active material	η_50_ (mV)	Tafel slope (mV dec^−1^)	R_ct_ Ω cm^2^	C_dl_ mFcm^−2^
(Mo/Co)O_x_–Cu@NF	410	276	0.028	53.4
CoO_x_–Cu@NF	440	145	0.072	37.2
Cu@NF	530	200	0.085	30.9
NF	613	298	0.100	1.57

13 $${{\text{C}}}_{{\text{dl}}}={{{\text{Y}}}_{{\text{dl}}}}^{1/{{\text{n}}}_{1}}\times {{({\text{R}}}_{{\text{s}}}^{-1}+{({{\text{R}}}_{{\text{ct}}}+{{\text{R}}}_{{\text{f}}})}^{-1})}^{({n}_{1}-1)/{n}_{1}}$$

It is obvious that (Mo/Co)O_x_–Cu@NF has the lowest R_ct_ value ~ 0.028 Ω cm^2^ and the highest C_dl_ value ~ 53.4 mF cm^−2^ compared to the other samples, confirming the best electrochemical catalytic activity during OER. Moreover, It is clear from Fig. S6b,d that (Mo/Co)O_x_ plays the greatest role versus metallic Cu for catalyzing OER. It gives almost the same η value as the ternary catalyst (410 mV at 50 mA cm^−2^) but the difference increase as the current density increases. So, one can conclude that the addition of metallic copper does not add much at lower current densities but it lowers the η value at high current densities which can attributed to the surface electron richness and high conductivity of metallic Cu^77^ versus the other individual Mo and Co oxides.

### Overall water splitting cell performance

**Figure 11:**
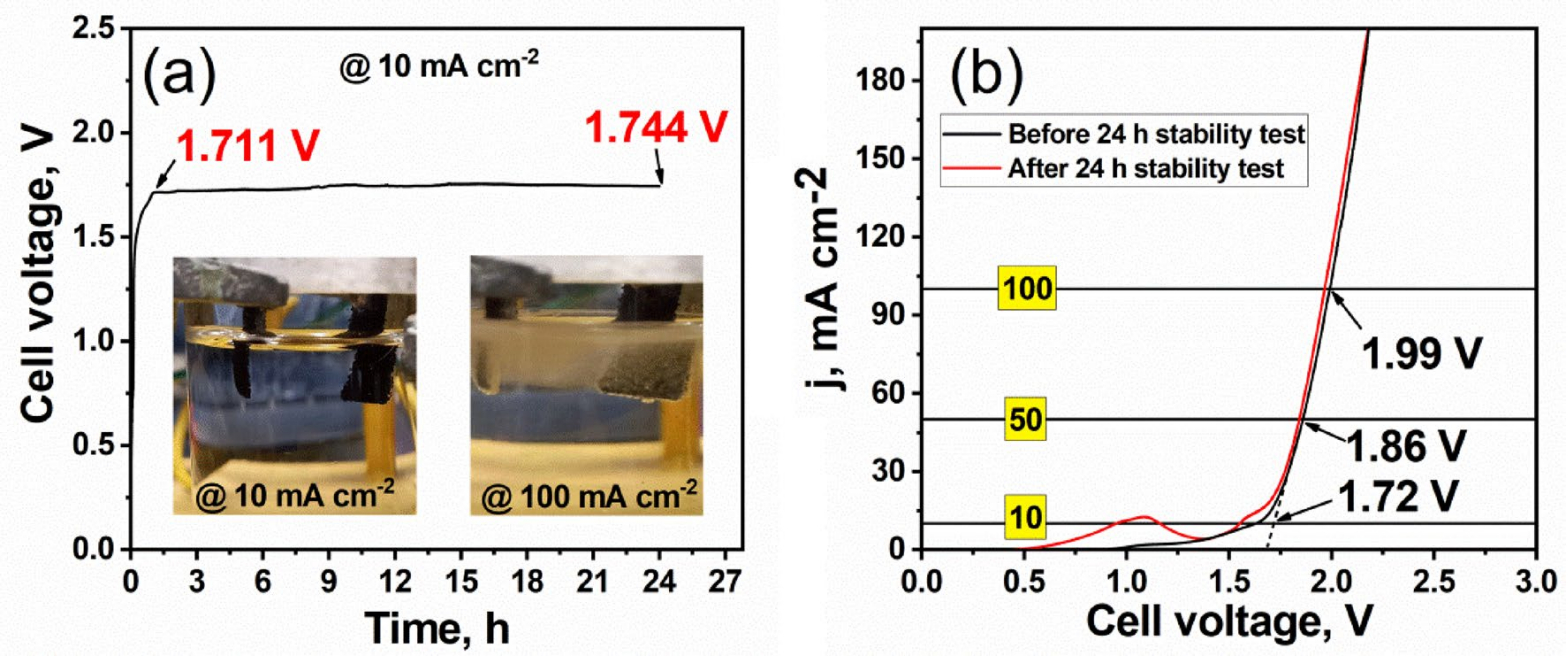
(**a**) overall water-splitting stability test of (Mo/Co)O_x_–Cu@NF measured in a symmetrical two-electrode cell using chronopotentiometry at 10 mA cm^−2^ for 24 h and (**b**) LSV for two (Mo/Co)O_x_–Cu@NF electrode-cell measured at 5 mV s^−1^ before and after the chronopotentiometric measurement for 24 h. All recorded in 1 mol L^−1^ KOH at 25 °C.

Stability of the prepared ternary (Mo/Co)O_x_–Cu@NF composite was assessed in a symmetrical two-electrode cell as shown in Fig. 11a using chronopotentiometry at 10 mA cm^−2^ for 24 h. It is clear that the ternary electrocatalyst is showing good stability where the cell voltage just increases by a value of 33 mV at the end of the 24 h test. Moreover, as shown in Fig. 11b LSV was measured at a scan rate of 5 mV s^−1^ for the symmetrical two-electrode cell of (Mo/Co)O_x_–Cu@NF. It was found that the cell needs 1.72 V, 1.86 V, and 1.99 V to give current densities at 10 mA cm^−2^, 50 mA cm^−2^, 100 mA cm^−2^, respectively. These values are comparable to the bench-mark commercial electrocatalysts Pt-C/NF(-)||RuO_2_/NF(+) based cells of values 1.58 V, and 2.07 V to give current densities at 10 mA cm^−2^, and 100 mA cm^−2^, respectively^82^.Interestingly, the LSV measured directly after 24 h of the chronopotentiometry test is nearly the same or better than that measured directly before the chronopotentiometry test. In Fig. 11b, the appearance of large peak around 1 V before the steep current rise corresponding to the overall water splitting after electrolysis stability test, may be attributed to three possible oxidation reactions for (Cu^1+^ to Cu^2+^), (Co^2+^ to Co^3+^), or (Mo^5+^ to Mo^6+^).The appearance of small peak at 1.62 V may correspond to (Cu^2+^ to Cu^3+^), (Co^3+^ to Co^4+^). Since the electrode where HER occurs, higher number of Co^3+^, and Mo^6+^ oxides centers become at lower oxidation states than that at the beginning of electrolysis stability test. So there are more Co, Mo oxides of lower oxidation states besides the metallic copper become susceptible to oxidation to higher oxidation states. Thus, the results indicate that the fabricated ternary composite (Mo/Co)O_x_–Cu@NF has satisfactory stability.

Table 4 introduces the electrocatalytic activity of our fabricated ternary catalyst in contrast to the reported ternary structures of similar components. Our fabricated ternary electrocatalystis of comparable performance to (Cu_x_Mo_x_/Co_1−x_NPs@RGO deposited on glassy carbon^83^ and Cu_x_O@NiO–MoO_2_ deposited on graphite^84^) with respect to OER. In addition, the η value for the same current density of our ternary electrocatalyst is similar to that achieved by the Co_56_Mo_21_Cu_23_ alloy deposited over carbon steel^48^ with respect to HER. Moreover, the catalytic activity of our ternary electrocatalyst is better than that of Co–Fe–Mo deposited over a copper sheet^74^ with respect to OWS.

**Table 4:** Comparison of the OER, HER, and OWS performances in 1 mol L^−1^ KOH of the prepared (Co_/_Mo)O_x_–Cu/NF with reported catalysts in liretature.

Electrocatalysts	Substrate	η_HER_ (mV) @(…) mA cm^−2^	η_OER_ (mV) @(…) mA cm^−2^	E_overall_(V) @(…) mA cm^−2^	Ref
Cu_x_Mo_x_/Co_1−x_NPs@RGO	Glassy carbon	–	390@50	–	^83^
CoCu/CuCoMoOx	Copper foam	75@100	315@100	1.66@100	^46^
Mo-doped Cu_x_Co_y_O_100_	Nickel foam	88@10	–	–	^47^
Co_56_Mo_21_Cu_23_alloy	Carbon steel	119@10	–	–	^48^
Cu_x_O@NiO-MoO_2_	Graphite	65@10	321@50	1.54@10	^84^
Co-Fe-Mo	Copper sheet	128@10	455@10	–	^74^
Co-Mo-PB	Glassy carbon	–	195@10	–	^85^
W, P-FeB	Nickel foam	–	209@10	–	^86^
(Co/Mo)O_x_–Cu	Nickel foam	188 @50	410@50	1.86@50	This work

## Conclusion

Briefly, a novel mixed heterovalent (Mo/Co)O_x_–Cu electrocatalyst system was chemically deposited on Ni foam through a stepwise solvothermal process. The physical, chemical, and functional analyses confirmed the formation of the hybrid ternary electrocatalyst of (Mo/Co)O_x_–Cu@NF with high homogeneity and uniform morphology. XPS investigation results revealed the presence of multiple oxidation states in the ternary system (Mo/Co)O_x_–Cu@NF, the property which is expected to enhance HER and OER in the alkaline medium. Engaging (Mo/Co)O_x_–Cu@NF in a two-electrode alkaline electrolyzer for a water-splitting reaction, a current density of 10 mA cm^−2^ can be attained at a cell voltage as low as 1.72 V with significant durable stability. The high catalytic performance with excellent stability arises from the combination of the amorphous structure with high surface area, and the crystalline structure of high electronic conductivity, as well as the richness of active sites provided by the haterovalent atoms. The work introduced a simple one-step method to fabricate a low-cost, efficient, and durable transition metal-based electrocatalyst which is noteworthy to be scaled up for bulk water electrolysis.

### Supplementary Information

Supplementary Information.

## Data Availability

All data presented in this study are included in this published article.
