# Supplementary material for: Novel mixed heterovalent (Mo/Co)Ox-zerovalent Cu system as bi-functional electrocatalyst for overall water splitting
Source: Sci Rep. 2024 Feb 26;14:4601. doi: 10.1038/s41598-024-54934-9 (PMC10897199; doi:10.1038/s41598-024-54934-9)
Supplement: Supplementary file 1 — Supplementary Information. [file 41598_2024_54934_MOESM1_ESM.docx]

**Novel mixed heterovalent (Mo/Co)O_x_-zerovalent Cu system as bi-functional electrocatalyst for overall water splitting**

**Ahmed R. Tartour^1,2^, Moustafa M.S. Sanad^1,^*, Ibrahim S. El-Hallag^3^, and Youssef I. Moharram^3,^***

*^1^Central Metallurgical Research & Development Institute, P.O. Box: 87 Helwan, 11421, Cairo, Egypt*

*^2^Electroplating Department, Factory 100, Abu-Zaabal Company for Engineering Industries, Cairo, Egypt*

*^3^Chemistry Department, Faculty of Science, Tanta University, Tanta, Egypt*

Preparation procedure for precursor solutions used to prepare the other metallic combinations-based electrocatalysts:

(Mo/Co)O_x_@NF

1 mmol fumaric acid (FA, 99%), and 0.1 mmol β-cyclodextrin (β-CD, 98%) were dissolved in 24 ml Dimethyl formamide (DMF, 99%) to form a clear solution (A). 1 mmol CoCl_2_.6H_2_O was separately dissolved in 6 ml absolute ethanol to form a clear solution (B).0.1 mmol of (NH_4_)_6_Mo_7_O_24_.4H_2_O was also dissolved in 6 ml deionized water to form a clear solution (C).Then, we simultaneously added solutions (B) and (C) into solution (A),and let the mixture under stirring for1 hour.

MoO_x_-Cu@NF

1 mmol fumaric acid (FA, 99%), and 0.1 mmol β-cyclodextrin (β-CD, 98%) were dissolved in 24 ml Dimethyl formamide (DMF, 99%) to form a clear solution (A). 1 mmol CuCl_2_.2H_2_O was separately dissolved in 6 ml absolute ethanol to form a clear solution (B). 0.1 mmol of (NH_4_)_6_Mo_7_O_24_.4H_2_O was also dissolved in 6 ml deionized water to form a clear solution (C).Then, we simultaneously added solutions (B) and (C) into solution (A),and let the mixture under stirring for1 hour.

CoO_x_-Cu@NF

1 mmol fumaric acid (FA, 99%), and 0.1 mmol β-cyclodextrin (β-CD, 98%) were dissolved in 24 ml Dimethyl formamide (DMF, 99%) to form a clear solution (A). 0.5 mmol CoCl_2_.6H_2_O,and 0.5 mmol CuCl_2_.2H_2_O were separately dissolved in 6 ml absolute ethanol to form a clear solution (B). Then, we simultaneously added solutions (B) and 6 ml deionized water into solution (A), and let the mixture under stirring for1 hour.

MoO_x_@NF

1 mmol fumaric acid (FA, 99%), and 0.1 mmol β-cyclodextrin (β-CD, 98%) were dissolved in 24 ml Dimethyl formamide (DMF, 99%) to form a clear solution (A). 0.1 mmol of (NH_4_)_6_Mo_7_O_24_.4H_2_O was also dissolved in 6 ml deionized water to form a clear solution (B).Then, we simultaneously added solutions (B) and 6 ml absolute ethanol into solution (A),and let the mixture under stirring for1 hour.

CoO_x_@NF

1 mmol fumaric acid (FA, 99%), and 0.1 mmol β-cyclodextrin (β-CD, 98%) were dissolved in 24 ml Dimethyl formamide (DMF, 99%) to form a clear solution (A). 1 mmol CoCl_2_.6H_2_O was separately dissolved in 6 ml absolute ethanol to form a clear solution (B). Then, we simultaneously added solutions (B) and 6 ml deionized water into solution (A),and let the mixture under stirring for1 hour.

Cu@NF

1 mmol fumaric acid (FA, 99%), and 0.1 mmol β-cyclodextrin (β-CD, 98%) were dissolved in 24 ml Dimethyl formamide (DMF, 99%) to form a clear solution (A). 1 mmol CuCl_2_.2H_2_O was separately dissolved in 6 ml absolute ethanol to form a clear solution (B). Then, we simultaneously added solutions (B) and 6 ml deionized water into solution (A),and let the mixture under stirring for1 hour.





**Figure S1.** FE-SEM images (a-d) showing the texture and morphology of the fabricated unary Cu@NF system at different magnifications.





**Figure S2.** (a) SEM image of the fabricated unary Cu@NF system and their corresponding (b) EDS spectrum and (c-e) elemental mapping.


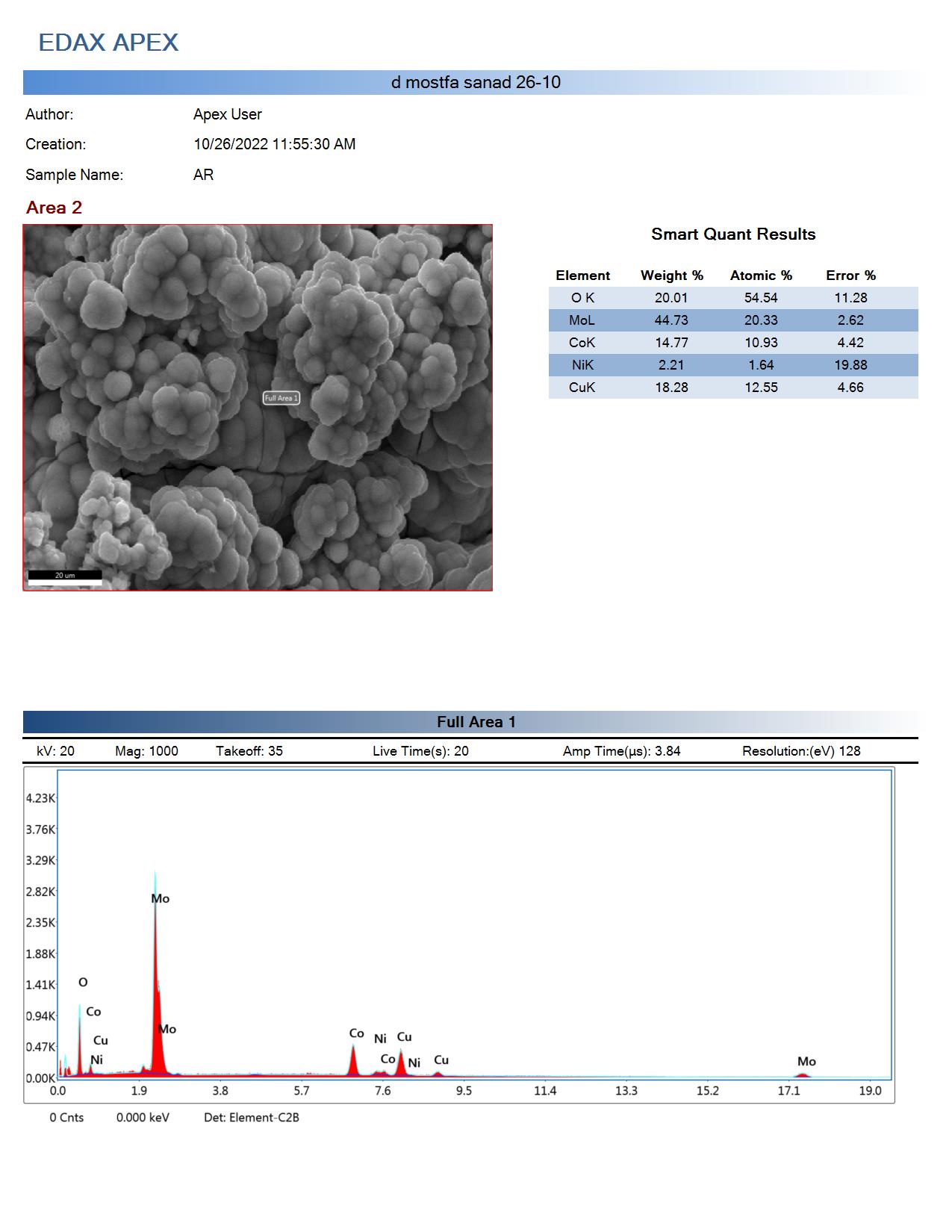


**Figure S3.** Raw data for EDS spectrum and elemental composition percentages including the small Ni percentage attributed to NF.





**Figure S4. (a) TEM image of the fabricated ternary (Mo/Co)O_x_-Cu@NF system, (b) HR-TEM, and (c) its corresponding SAED.**


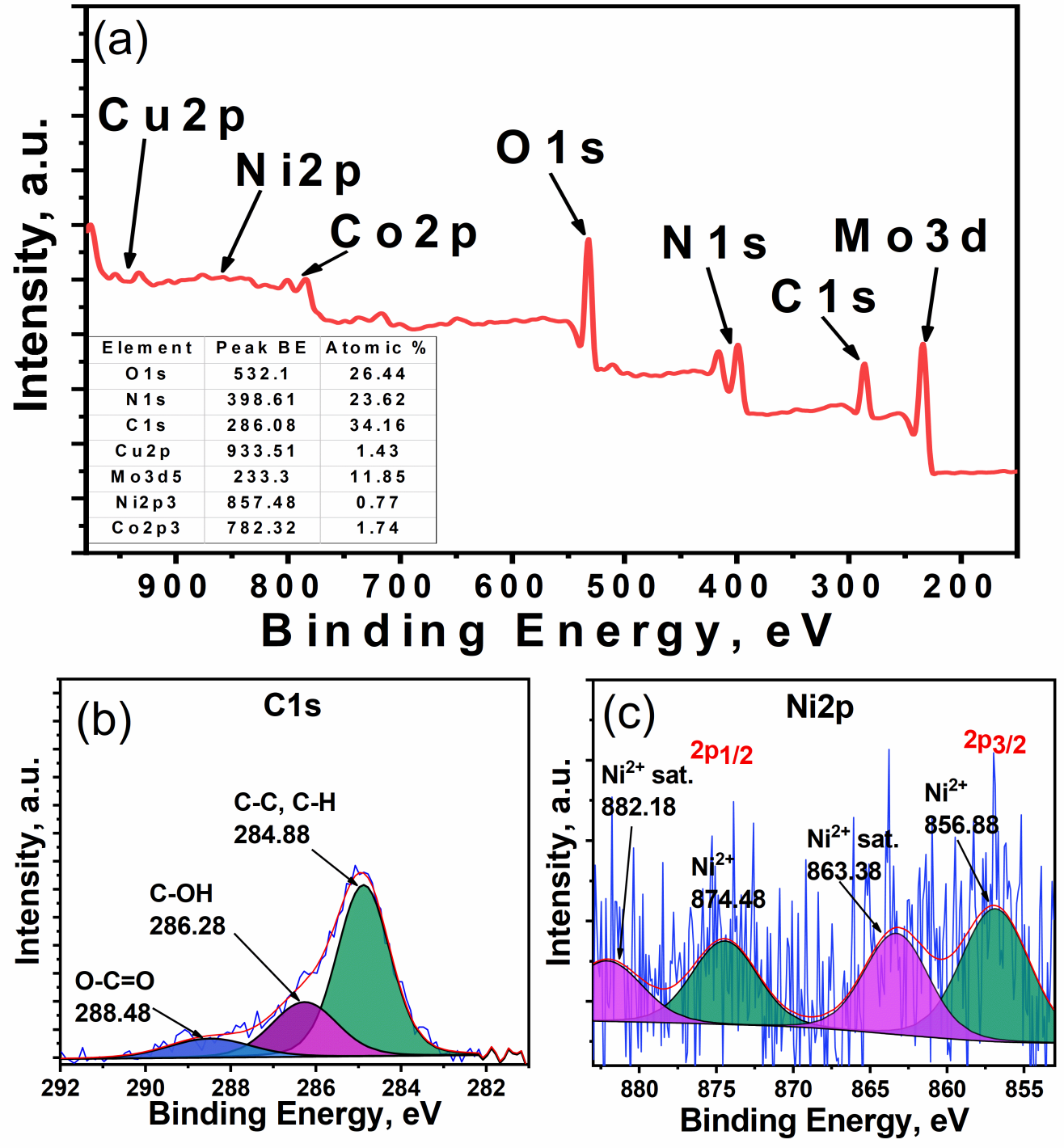


**Figure S5.** (a) XPS survey for (Mo/Co)O_x_-Cu@NF system and the detailed spectra of (b) C1s, and (c) Ni2p.


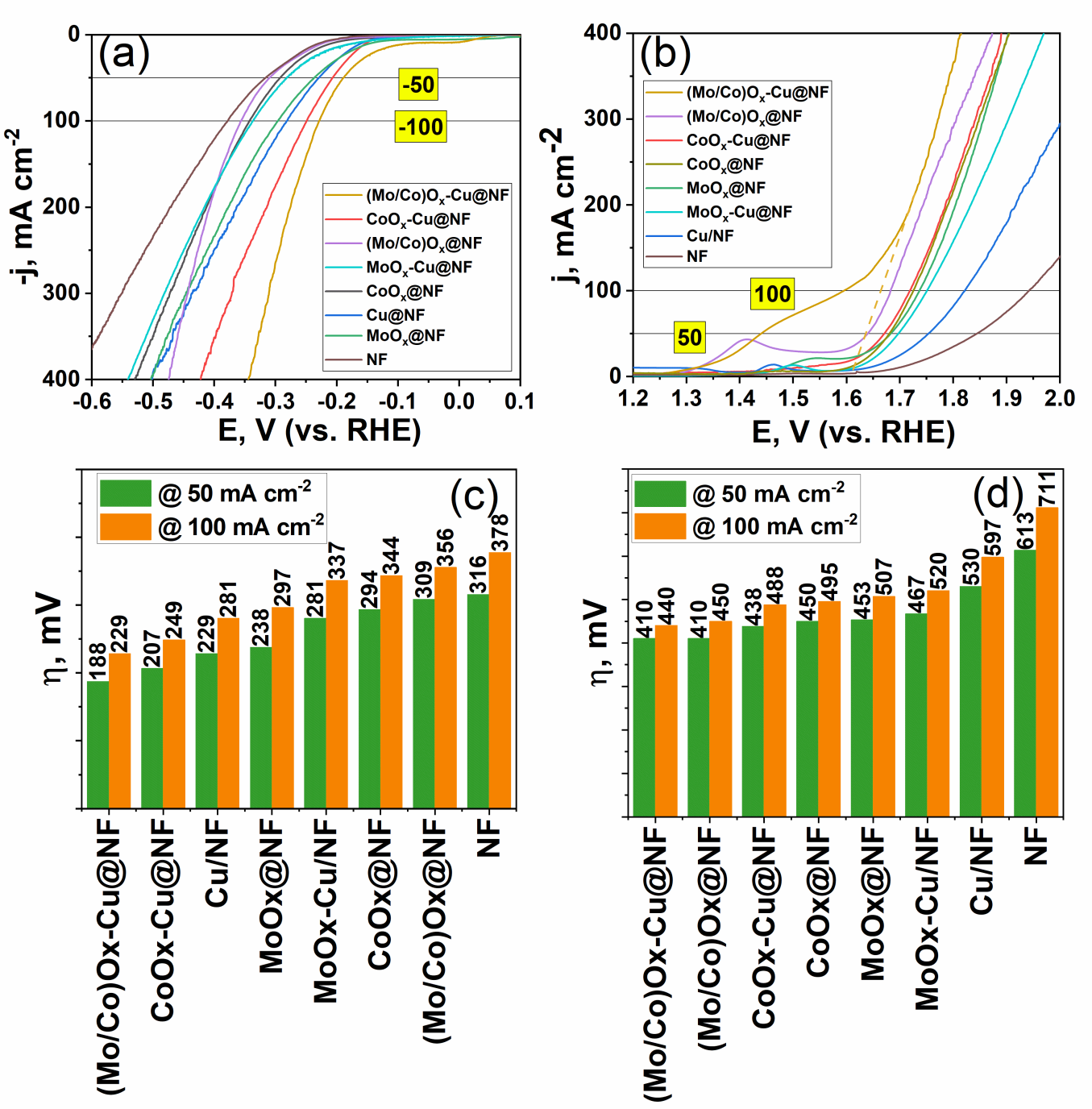


**Figure S6.** Linear sweep voltammetric curves for (a) HER and (b) OER and (c) and (d) column graph for the corresponding overpotentials estimated at 50 and 100 mA cm^-2^. All measurements recorded in 1 mol L-1 KOH, at 25°C.

**Table S1** Fitting data extracted from EIS For HER for the prepared catalytic materials

| Active material | R_s_  Ω cm^2^ | R_ct_  Ω cm^2^ | Y_dl_  mF s^α-1^cm^-2^ | n | C_dl_  mF cm^-2^ |
| --- | --- | --- | --- | --- | --- |
| (Mo/Co)O_x_-Cu@NF | 2.01 | 1.29 | 48.95 | 0.71 | 217 |
| CoO_x_-Cu@NF | 1.76 | 1.55 | 4.225 | 0.83 | 5.46 |
| Cu@NF | 2.07 | 3.46 | 2.823 | 0.85 | 3.55 |
| NF | 2.50 | 3.08 | 0.049 | 1.01 | 0.05 |

**Table S2** Fitting data extracted from EIS For OER for the prepared catalytic materials

| Active material | R_s_  Ω cm^2^ | R_ct_  Ω cm^2^ | Y_dl_  mF s^n-1^cm^-2^ | n_1_ | R_f_  Ω cm^2^ | Y_f_  mF s^n-1^cm^-2^ | n_2_ | C_dl_  mF cm-2 |
| --- | --- | --- | --- | --- | --- | --- | --- | --- |
| (Mo/Co)O_x_-Cu@NF | 1.95 | 0.012 | 53.38 | 1.00 | 0.39 | 1450 | 0.55 | 53.4 |
| CoO_x_-Cu@NF | 1.71 | 0.072 | 23.15 | 0.83 | 0.52 | 81.78 | 0.78 | 37.2 |
| Cu@NF | 2.03 | 0.085 | 18.79 | 0.84 | 1.07 | 34.78 | 0.84 | 30.9 |
| NF | 2.28 | 0.100 | 1.562 | 0.98 | 1.19 | 10.26 | 0.83 | 1.57 |

.
